# Dream Recall Frequency Is Associated With Medial Prefrontal Cortex White-Matter Density

**DOI:** 10.3389/fpsyg.2018.01856

**Published:** 2018-09-27

**Authors:** Raphael Vallat, Jean-Baptiste Eichenlaub, Alain Nicolas, Perrine Ruby

**Affiliations:** Lyon Neuroscience Research Center, Brain Dynamics and Cognition Team (DYCOG), INSERM UMRS 1028, CNRS UMR 5292, Université Claude Bernard Lyon 1, Université de Lyon, Lyon, France

**Keywords:** dreaming, dream recall, voxel-based morphometry, sleep, MRI, hippocampus, amygdala, DMN

## Abstract

Recent findings indicate that dream recall frequency (DRF) is associated with neurophysiological traits, and notably the regional cerebral blood flow at rest within the medial prefrontal cortex (MPFC) and the temporo-parietal junction (TPJ). To test whether, such physiological traits are rooted in anatomical specificities, we used voxel-based morphometry to compare the white matter and gray matter density in regions related to dream recall (either at the experimental or theoretical level, MPFC, TPJ, hippocampus and amygdala) between 46 high dream recallers (HR, DRF = 5.98 ± 1.25 days per week with a dream report) and 46 low dream recallers (LR, DRF = 0.34 ± 0.29). We found an increased medial prefrontal cortex white-matter density in HR compared to LR but no other significant difference between the two groups. These results are consistent with previous studies showing that lesions within the white matter of medial prefrontal cortex are associated with a partial or total cessation of dream reporting and suggest an implication of this region in dream recall or, more likely, in dream production.

## Introduction

While dreaming has long been considered as the cognitive correlate of rapid eye movement (REM) sleep, it is now well established that dreaming can occur in any sleep stage and is therefore not exclusive to a specific sleep stage (Solms, 2000; Nir and Tononi, 2010; Ruby, 2011; Montangero, 2018). It is consequently currently out of reach to know for sure when one is actually dreaming while asleep (i.e., no physiological marker of dreaming has been discovered yet). For that reason, most empirical investigation of dreaming (be it the investigation of dream content, or frequency) are based on the study of dream memories reported after the awakening of the dreamer (e.g., Schwartz and Maquet, 2002; Fosse et al., 2003; Schredl et al., 2003; Schwartz, 2003; Zadra and Robert, 2012; Windt, 2013; Vallat et al., 2017a). Studies on dreaming have thus highlighted the cognitive and cerebral correlates of dream recall, either by investigating the EEG in the minutes preceding an awakening followed (or not) by a dream recall (Esposito et al., 2004; Wittmann et al., 2004; Chellappa et al., 2011; Marzano et al., 2011; Scarpelli et al., 2015; Siclari et al., 2017), or by evaluating the cognitive and brain functioning associated with high and low DRF using behavioral methods, EEG or fMRI (Ruby, 2011 for a review; Eichenlaub et al., 2014a,b; Vallat et al., 2017b, 2018a).

Using this last strategy, recent works from our team highlighted several neurophysiological differences between HR (more than 3 days per week with a dream recall) and LR (less than 2 days per month with a dream recall), not only during sleep but also during wakefulness (Eichenlaub et al., 2014a,b; Vallat et al., 2017b). Notably, using PET, we compared the spontaneous rCBF of HR and LR during sleep and wakefulness, and showed that HR have a higher spontaneous rCBF than LR in the temporo-parietal junction (TPJ) and in the MPFC during REM sleep, N3 sleep and wakefulness (Eichenlaub et al., 2014b).

We concluded that these two regions played a key role in dream production or recall since lesions of these same areas have been found to be consistently associated with global or partial cessation of dream reporting (without any concurrent sleep disturbance; see Solms, 1997, 2000).

To our knowledge, only one study has so far investigated the relationship between brain anatomy and dream recall (De Gennaro et al., 2011). In this study, the authors used multiple regression analysis to evaluate the linear relationships between measures of some deep gray matter structures (amygdala and hippocampus) and quantitative and qualitative aspects of dream reports. They reported that the neuroanatomical measures were not associated with the number of dreams recalled per day, but they were with some features of dream reports such as length, emotional load, bizarreness, and vividness.

In the present study, we intended to further test the possible association between brain anatomical structures and dream report frequency. In line with previous results, we targeted brain regions previously associated with dream recall at the experimental or theoretical level (MPFC & TPJ, Solms, 1997; Eichenlaub et al., 2014b; hippocampus and amygdala, Maquet et al., 2005; Levin and Nielsen, 2007; De Gennaro et al., 2011; Perogamvros and Schwartz, 2012; Llewellyn, 2013; Malinowski and Horton, 2015). We expected that HR would show an increased density of the gray-matter and/or white-matter in the MPFC and/or the TPJ but would not show such an increase in the hippocampus and the amygdala.

## Methods

### Participants

Data for this study comes from two distinct neuroimaging studies (Eichenlaub et al., 2014b; Vallat et al., 2018b). In both studies, the main inclusion criterion was self-reported habitual weekly DRF, assessed by questionnaires (Vallat et al., 2018a). The inclusion criteria for HR were at least 3 days per week with a dream recall, and for LR, at most 2 days per month with a dream recall. No subjects had a history of medical, neurological, or psychiatric disease or was on medication at the time of the studies. The subjects provided written informed consent according to the Declaration of Helsinki. The studies were approved by the local ethics committee (CCPPRB, Centre Leon Berard, Lyon, France), and the subjects were paid for participation.

The anatomical scans acquired during wakefulness of 92 participants were analyzed (mean age = 22.45, standard deviation = 2.23, range = 19–29). Among them, 46 were HR and 46 were LR. As can be seen in **Table 1**, the two groups differed significantly in DRF, but not in age, sex ratio and body mass index (BMI).

**Table 1:** Group demographics. *P*-values were obtained using two-sided *t*-tests for age, DRF, and BMI and using chi-square for the sex ratio.

	*n*	Age	Sex ratio (M/F)	Habitual Weekly DRF	BMI
Mean HR	46	22.52	2.1	5.98	22.63
*STD HR*	*–*	*2.22*	*–*	*1.25*	*2.36*
Mean LR	46	22.38	2.8	0.34	22.45
*STD LR*	*–*	*2.26*	*–*	*0.29*	*2.50*
*p-value*	*–*	*0.77*	*0.49*	*<0.001*	*0.71*

### MRI Data Collection

Structural data for both studies were acquired at the CERMEP neuroimaging facility in Lyon, France. In Study A (21 HR and 20 LR) (Eichenlaub et al., 2014b), structural MRI were acquired with a T1-weighted MPRAGE sequence (1.0 mm isotropic resolution) on a 1.5T Siemens Sonata MR system (Siemens Medical Solutions, Erlangen, Germany). In Study B, structural MRI were acquired with a T1-weighted MPRAGE sequence (0.9 mm isotropic resolution) on a 3T Siemens Primage MR system (28 HR and 27 LR, Vallat et al., 2018b). Out of the 96 subjects, four were excluded based on age or BMI outlier rejection.

### VBM Analyses

#### Preprocessing

Prior to preprocessing, all raw images were visually inspected for potential artifacts. Data preprocessing was performed using the CAT12 toolbox^1^ (Gaser and Dahnke, 2016) for SPM12 (Wellcome Department of Cognitive Neurology). Data were normalized to MNI stereotactic space using DARTEL registration (Ashburner, 2007), corrected for bias field inhomogeneities, and segmented into gray matter, white matter, and cerebrospinal fluid. The mean correlation and weighted overall image quality were then computed and visually plotted to perform quality check and outlier rejection. The mean correlation measures the homogeneity of the data after pre-processing, whereas the weighted overall image quality combines measurements of noise and spatial resolution of the image before preprocessing. Four participants were excluded from further analyses because of a low data quality (one HR and one LR from Study A, one HR and one LR from study B). For the remaining 88 participants, we estimated the total intracranial volume (TIV) to further correct for different head size and volume. Images were then smoothed with an isotropic Gaussian kernel of 6 mm full width at half-maximum.

#### Statistical Design

The normalized modulated smoothed white and gray matter images were entered into an independent two sample *T*-test statistical model, together with age and TIV as covariates. We restricted the analyses to the MPFC, TPJ, hippocampus and amygdala (**Supplementary Figure S1**). A single binary spatial mask comprising these four regions was created by combining the individual and thresholded spatial masks of each of these regions (using the *fslmaths* command). The individual spatial masks were defined by generating a map in Neurosynth^2^, using the keywords ‘amygdala,’ ‘hippocampus,’ ‘medial prefrontal,’ and ‘temporoparietal junction,’ respectively. The four maps were then thresholded at *z* = 7 to increase anatomical specificity. The significance threshold was set at *p* < 0.001 uncorrected with an extent threshold of 10 voxels.

## Results

**FIGURE 1:**
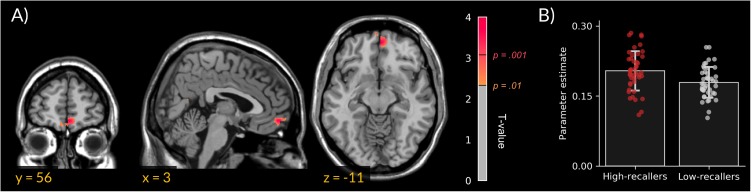
Increased medial prefrontal cortex white-matter density in high dream recallers. **(A)** Statistical parametric map showing an increased white-matter density in HR (*N* = 44) as compared to LR (*N* = 44; peak cluster in MNI coordinates = 3, 56, –11). For display purpose, threshold was set at *p* < 0.01 uncorrected. **(B)** Bar plots of cluster-averaged parameter estimate in both groups. Points represent individual values.

We did not find gray matter density differences between HR and LR. However, VBM analyses revealed an increased MPFC white-matter density in HR compared to LR (peak cluster in MNI coordinates = 3, 56, 11; extent = 172 voxels; **Figure 1**).

## Discussion

This study intended to test whether an increased DRF in HR could be associated with gray- or white- matter density specificities in brain regions previously associated (at the theoretical or experimental level) with dream recall and/or production, namely the amygdala, hippocampus, MPFC and TPJ. VBM analyses of the anatomical scans of 44 HR and 44 LR revealed a significant difference between the two groups in the white matter of the MPFC. This result adds an anatomical dimension to numerous experimental findings showing differences in brain functioning between HR and LR (Eichenlaub et al., 2014a,b). To the best of our knowledge, this is the first study reporting brain structural differences between HR and LR.

The absence of a significant group difference in the amygdala and hippocampus replicate previous results (Torda, 1969; De Gennaro et al., 2011) and support the idea that these two regions are not directly involved in dream memory frequency, even if they might be involved in some qualitative aspects of dream content (Torda, 1969; Maquet and Franck, 1997; Revonsuo, 2000; Maquet et al., 2005; Nielsen and Stenstrom, 2005; De Gennaro et al., 2011; Solms, 2013; Corsi-Cabrera et al., 2016).

The significant group difference in the white-matter of the MPFC is well in line with previous neuropsychological findings showing a cessation of dream reports after lesion in the white matter surrounding the frontal horns of the lateral ventricles (Solms, 1997, 2000). To explain this observation, Solms stressed that the ventromedial prefrontal cortex contains a substantial number of fibers connecting frontal and limbic structures with dopaminergic cells in the ventral tegmentum. He further suggested that dreaming is generated by this dopamine circuit, i.e., that the mesocortical-mesolimbic dopamine system plays a causal role in the generation of dreams (Solms, 2000). Our results support this conclusion by showing in healthy subjects a significant association between DRF and the white matter density in the MPFC. To assess whether it is an acquired or an innate characteristic, and to better understand the functional significance of a white matter density increase, future studies may measure the white matter density in the MPFC before and after an increase of DRF induced by an increase in attention to, or interest in dreams (as can be done with a dream diary, Schredl, 2002; Ruby, 2011).

At the functional level, some EEG studies have produced results compatible with an involvement of MPFC in dream recall (e.g., a positive association between increased frontal theta EEG power and successful dream recall, Marzano et al., 2011; Scarpelli et al., 2015) and a PET study has demonstrated an increased cerebral blood flow in the MPFC during sleep in HR as compared to LR (Eichenlaub et al., 2014b). This last result suggests that MPFC plays a role in dream production rather than in dream recall due to its tonic activity during sleep. In addition, previous results did not noticeably involve MPFC in memory recall. Rather, this region is involved in social cognition such as mind reading (Legrand and Ruby, 2009 for a review), social emotions processing (e.g., Ruby and Decety, 2004) and projective imagery (i.e., envisioning the future or the past, Buckner and Carroll, 2007). As such, it could participate in the production of the scenario or plot of the dream. This is consistent with results showing a similar level of rCBF in the MPFC during wake and REM sleep and an important drop of activity in this region during N3 sleep, a sleep stage associated with less dream reports than REM sleep (Nielsen, 2000; Maquet et al., 2005; Ruby, 2011). This interpretation is well in line with recent review articles arguing that dreaming is an intensified form of mind wandering and that at least part of the DMN of the brain (of which the MPFC is a core component, Gusnard and Raichle, 2001; Raichle et al., 2001) is involved in the production of dreams (Domhoff, 2011; Domhoff and Fox, 2015; Christoff et al., 2016). The DMN is a set of functionally coupled brain regions especially during internally oriented mental processes and episodic memory retrieval (Gusnard and Raichle, 2001; Raichle et al., 2001; Legrand and Ruby, 2009). This network is centered on the MPFC, the posterior cingulate cortex (PCC) and the lateral parietal areas around the TPJ area but also comprises the temporal pole and the hippocampus and parahippocampal gyrus (Legrand and Ruby, 2009; Raichle, 2015).

Finally, it should be noted that in this study, we investigated the possible brain anatomical correlates of the trait component of DRF. Yet, DRF has both trait and state components (Schredl and Reinhard, 2008; Ruby, 2011; Eichenlaub et al., 2014a,b). The state components of DRF mediated by, for example, sleep stage, time of night, the pre-sleep mood, or psychotropic drug use (Stickgold et al., 2001; Schredl, 2007; Nir and Tononi, 2010; Ruby, 2011; Tribl et al., 2013) most probably has functional rather than anatomical neurophysiological correlates (e.g., Esposito et al., 2004; Chellappa et al., 2011; Marzano et al., 2011; Scarpelli et al., 2015).

The main finding of this article is that, as compared to LR, HR show an increased white matter density in the MPFC. Altogether with previous functional (PET, EEG) and neuropsychological (lesions) results, our finding argues for a role of the MPFC in dream production.

## Author Contributions

AN, J-BE, RV, and PR participated in designing the study and collecting data. RV conducted the data analysis and wrote the first draft of the paper. PR participated in the writing of the article.

## Conflict of Interest Statement

The authors declare that the research was conducted in the absence of any commercial or financial relationships that could be construed as a potential conflict of interest.

## Footnotes

1 http://www.neuro.uni-jena.de/cat/

2 http://neurosynth.org/

## Supplementary Material

FIGURE S1 Spatial mask used in the VBM analysis. MPFC, medial prefrontal cortex. TPJ, temporoparietal junction.

## Abbreviations

BMI: body mass index
DMN: default mode network
DRF: dream recall frequency
EEG: electroencephalography
fMRI: functional magnetic resonance imaging
HR: high dream recallers
LR: low dream recallers
MPFC: medial prefrontal cortex
PET: positron emission tomography
rCBF: regional cerebral blood flow
TPJ: temporoparietal junction
VBM: voxel-based morphometry
