# Supplementary figures and images for: Dream Recall Frequency Is Associated With Medial Prefrontal Cortex White-Matter Density
Source: Front Psychol. 2018 Sep 27;9:1856. doi: 10.3389/fpsyg.2018.01856 (PMC6171441; doi:10.3389/fpsyg.2018.01856)

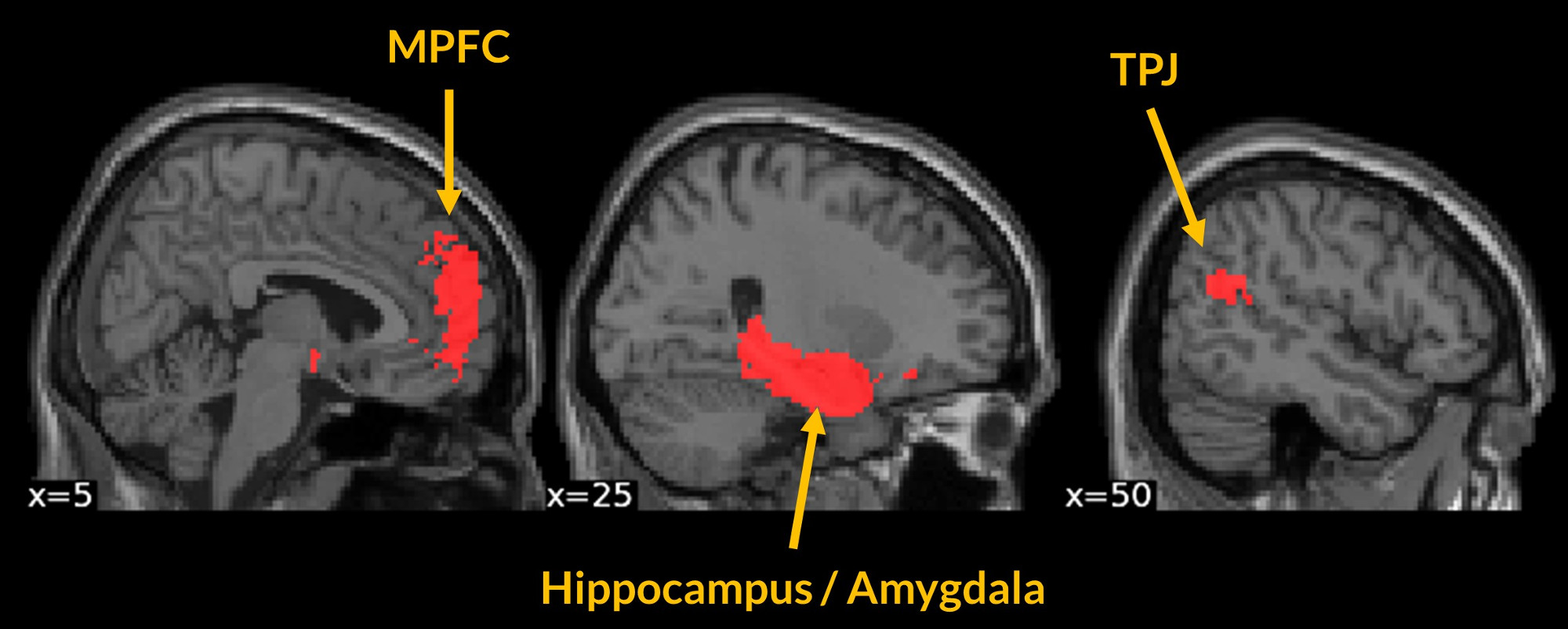

Supplement: FIGURE S1 — Spatial mask used in the VBM analysis. MPFC, medial prefrontal cortex. TPJ, temporoparietal junction. [file Image_1.JPEG]
